# Healthcare Professionals’ Perspectives of Patients’ Experiences of the Self-Management of Type 2 Diabetes in the Rural Areas of Pakistan: A Qualitative Analysis

**DOI:** 10.3390/ijerph18189869

**Published:** 2021-09-19

**Authors:** Rashid M. Ansari, Mark Harris, Hassan Hosseinzadeh, Nicholas Zwar

**Affiliations:** School of Public Health and Community Medicine, Faculty of Medicine, University of New South Wales, Sydney, NSW 2052, Australia; m.f.harris@unsw.edu.au (M.H.); hassanh@uow.edu.au (H.H.); n.zwar@unsw.edu.au (N.Z.)

**Keywords:** self-management, type 2 diabetes mellitus, methodological approach, middle-aged population, patient-doctor relationship, qualitative research

## Abstract

The main objective of this research work was to explore the healthcare professionals’ perspectives of type 2 diabetes patients’ experiences of self-management of diabetes in the rural area of Pakistan. In this study, we have carried out a methodological approach to use a self-management framework to direct the interview guide for healthcare professionals to examine their perceptions and expectations of their diabetes patients’ adherence to the medications prescribed. Twenty healthcare professionals were recruited in this study consisting of ten general practitioners and ten nurses from various clinics (medical centres) of Al-Rehman Hospital at Abbottabad, Pakistan. This qualitative study explored the feelings and opinions of general practitioners on patients’ compliance and adherence by using the semi-structured interview guide using a methodological framework. All interviews of participants were audiotaped and transcribed for content analysis. Six major themes were identified: patient–doctor relationship; patient’s non-adherence to diet and exercise; conflicts with the patients; low self-efficacy and feeling of “resignation with poor care”; the influence of culture on patients’ self-management activities and lack of support for patients by health care providers, patients, and their families. We have derived relevant solutions from qualitative studies and considered that communication, tailored, and shared care is the best approach for patient adherence to treatment. GPs felt that a structured consultation and follow-up in a multidisciplinary team might help to increase adherence. The results of this qualitative health research highlighted the challenges healthcare professionals are facing in rural Pakistan in managing patients with type 2 diabetes and supporting their management activities. Healthcare professionals and patients may benefit by adopting a methodological framework approach to ensure meaningful participation and adjusting the patient–doctor relationship, and setting up achievable management and self-management goals.

## 1. Introduction

The management of diabetes relies on the active self-management by patients [1]. The disease requires patient commitment and the ability to carry out self-management activities in their routine life [2]. The current literature review identified that diabetes self-management is the cornerstone of diabetes care [3]. Several studies [4,5] supported the concept that diabetes self-management is associated with improved diabetes knowledge, self-management behaviours, and clinical outcomes [6]. Fisher et al. [7] also suggested that quality clinical care and self-management are compatible and dependent on each other.

To self-manage, patients need to have awareness and knowledge about diabetes, its management, and its complications [8,9]. This entails adherence to a healthy diet, physical activity, self-monitoring of blood glucose, and adherence to prescribed medicines [10].

A cross-sectional study carried out in Pakistan by Bukhsh et al. [1] identified the important role of self-management activities. The authors evaluated the predictive relationship of diabetes-related self-management activities and demonstrated that the level of glycated haemoglobin (HbA1c) decreased as self-management activities increased.

In the context of self-management of type 2 diabetes, general practitioners’ perceptions of diabetes patients and their overall conditions have been identified to have significant influence on the course of a consultation [9,11]. Therefore, practitioners’ behaviour (in relation to doctor–patient communication) may be influenced by the general practitioners’ perspectives towards patients’ diabetes self-management activities [3,12].

The patient–doctor relationship [13] is an important aspect with great significance in the self-management of type 2 diabetes. Successful diabetes management requires teamwork approach between the doctor and the patient—that is, good communication and shared decision making between doctor and patient. Patients with diabetes need to engage with a range of health professionals in order to develop and participate in self-management activities [14].

The communication style of the general practitioners and techniques exert its effects on the health outcome of the patients [4]. For example: the general practitioner communicates different treatment plans to the patient and describes the risks and benefits of these plans. Then the patient expresses preferences for treatment to ensure that the disease management plan provided aligns with the patient’s values and needs [15,16].

A number of studies have also supported the ideas that patients’ understanding and beliefs about health and illness are heavily dependent on the historical and local contexts [14,15]. In addition, personal experience and observation of patient play an important role [16]. The previous research provides evidence of how the culture, tradition, and lifestyle activities of the middle-aged population of Pakistan influence the adherence of patients with diabetes to recommendations of general practitioners on healthy diet and physical activity [17,18,19]. These attitudes and behaviours pose significant challenges to self-management activities in that region.

Cultural and religious barriers are frequently cited as challenges [17,18,19,20]. The cultural and religious barriers affect the implementation of diabetes self-management activities [20]. Psychosocial barriers such as diabetes distress, pessimistic attitudes, lack of family and social support, and lack of willingness to change the behaviour influence longer-term outcomes, such as glycaemic control and eventually the development of diabetes complications [20]. A review of related literature [17,18,19,20,21,22,23,24,25] revealed that non-compliance with healthy eating requirements, lack of physical activity, lack of family and cultural support, and difficulties in accessing medical care are some of the barriers to self-management of diabetes [26].

The aim of this study was to explore healthcare professionals’ perspectives of type 2 diabetes patients’ experiences of self-management of diabetes in the rural area of Pakistan and to better understand differences in diabetes self-management among men and women living with type 2 diabetes mellitus. The other objectives of this study were to elicit problems physicians encounter with type 2 diabetes patients’ adherence to treatment recommendations; to search for solutions acceptable to patients and to find a better approach to sort out the problem in case of frustration.

## 2. Methods

### 2.1. Qualitative Design

The qualitative design of this study was guided by a methodological framework approach and the results of the systematic review of self-management of type 2 diabetes [27,28]. This framework described the healthcare professionals’ perspectives of type 2 diabetes patients and identified six major themes. With a qualitative methodological framework approach (used during interviews of healthcare professionals), this study aimed to help understanding the healthcare professional’s perspectives of patients with type 2 diabetes, experiencing difficulties with self-management activities and the barriers and challenges diabetes self-management is inflicting on these patients.

This qualitative design used consolidated criteria for reporting qualitative research (COREQ), and reported important aspects of the research team, study design, study context, findings, analysis, and interpretations of the results. The reporting technique such as COREQ is expected to improve the quality of reporting this research on healthcare professionals’ perspectives on type 2 diabetes mellitus [29].

**Figure 1:**
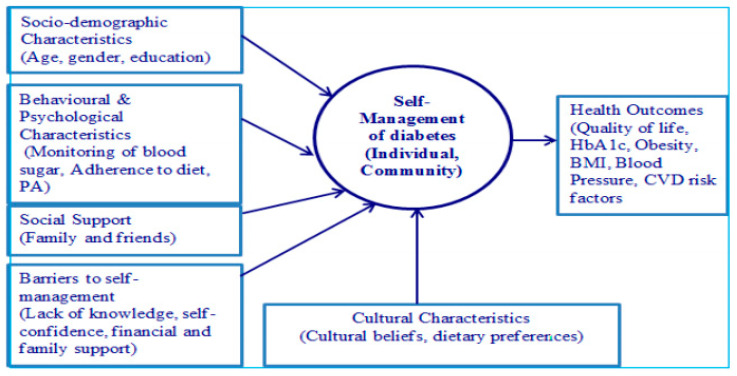
Conceptual model of self-management of type 2 diabetes [28].

The qualitative design was carried out using a methodological approach to use a self-management framework [27] to direct the interview guide developed for healthcare professionals and guided by the results of a systematic review of self-management of type 2 diabetes carried out by the authors [28]. Figure 1 provides a conceptual model of self-management of type 2 diabetes. This model explores the relationships between the variables that influence the self-management of type 2 diabetes among the middle-aged population of Pakistan [30].

This model provides valuable information to healthcare professionals that socio-demographic characteristics, behavioural and psychological characteristics, social support, barriers to self-management, and cultural characteristics impact diabetes self-management. Therefore, healthcare professionals’ perspectives should be to improve upon these factors as these factors combined together predict health outcomes. In Pakistani culture, strong family bonds are important and highly valued [31]. Therefore, medical treatment and decisions by general practitioners should be made keeping the family and cultural set up in mind rather than by the discretion of an individual [19,20,21].

This methodological approach is unique and first of its kind which describes the activities related to diabetes self-management as a series of various tasks such as diet and exercise, blood glucose monitoring, medication adherence, and consultation with general practitioners. According to Creswell [32], this type of qualitative study approach allows for rich, in-depth understanding through a holistic framework and, therefore, most suited to answer the following research questions in this study.

- What are the differences between physician and patient understandings of self-management of type 2 diabetes in the rural area of Pakistan?
- What factors affect diabetes self-management practices?

### 2.2. Sample and Recruitment

The recruitment of healthcare professionals was carried out from various primary health care centres of Al-Rehman Hospital in the rural area of Abbottabad. A total 40 healthcare professionals were invited from various clinics in that area and the selection was made by the medical director based on their experiences managing the patients of type 2 diabetes. The participant’s selection was based on selective sampling (purposive sampling) approach as it allows to select the participants based on a specified criterion in the sample [33].

The 20 healthcare professionals consisted of 10 general practitioners and 10 nurses were selected. The remaining 10 healthcare professionals were dropped as they were not meeting the minimum 10 years’ experience of type 2 diabetes management. All the nurses were female as the profession of nursing is only reserved for females in that region. Among the total 20 participants (10 general practitioners: 5 males and 5 females and 10 female nurses), the average age was 50 years with an average of 15 years in clinical practice.

### 2.3. Consent to Participate

The participants were provided written consent in the form of an “Information Sheet” and a consent form before the actual interviews were carried out. The English version of the information sheet and consent form was translated into participants’ native language particularly for nurses who do not speak the English language. Since the researcher speaks the same language, an interpreter was not required.

### 2.4. Data Collection

The interviews with 20 healthcare professionals (10 general practitioners and 10 nurses) were conducted face-to-face in the Urdu language in a clinical setting at various medical centres of Al-Rehman hospital, Pakistan. The interview lasted 40–50 min. The semi-structured interviews were transcribed and translated from Urdu language. Following an initial check for completion, data collection, all identifying information of the participants was removed and objective identifiers were used on the transcripts to ensure anonymity. The qualitative interview guidelines for healthcare professionals are provided in Appendix A.

### 2.5. Qualitative Data Analysis

**Figure 2:**
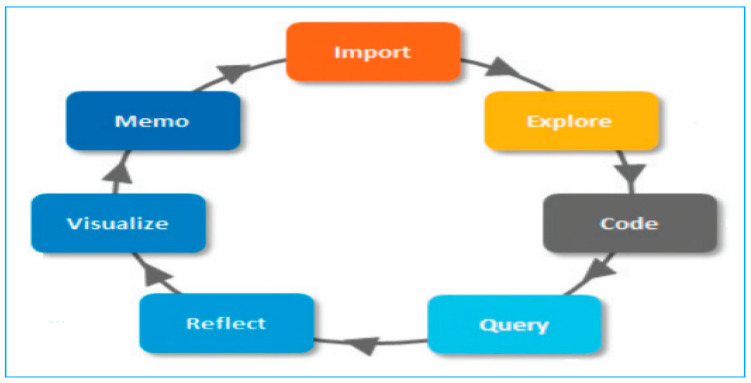
The qualitative analysis path to explore the themes.

The transcripts were imported into NVivo 11 Pro-QSR International [34] and thematically analysed [35,36]. The research team followed the quality framework outlined by Meyrick [37] and a methodological approach to use a self-management framework proposed by Brewer-Lowry [27]. In our research team, two academics participated in the analysis with background in qualitative analysis. Figure 2 shows the path adopted when we have explored the themes for the detailed analysis. The figure highlights various steps to carry out the analysis in NVivo 11 Pro, from importing the interview documents to exploring, visualizing, and reflecting upon it and finally providing the codes.

The thematic analysis involved initial independent coding by the two academics and the researcher who identified several codes. The codes were reviewed and then clustered and used to form preliminary sub-themes that integrated several of the originally identified codes and encompassed more general topics that were the focus of the transcripts. Difference of opinion was resolved through discussions between the researcher and the academics and the local expert until consensus was reached. The researcher and academics reached to the point of saturation where they did not obtain any new theme and new code. They have applied different perspectives during the data analysis so that the validity of the results was ensured [38].

## 3. Results

**Figure 3:**
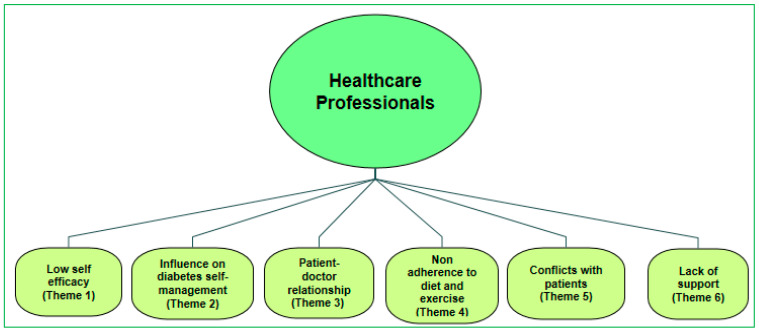
Themes from healthcare professionals’ perspectives of patients’ experiences of self-management of type 2 diabetes.

The summary of participants’ demographics is provided in Table 1. There were a total of 20 participants and the average age of the participants was 49 years, 10 general practitioners and 10 female nurses with an average of 15 years in clinical practice. The thematic analysis was initially involved independent coding by two academics. Total fifty codes were identified in the preliminary analysis of the transcripts and codes were then clustered and used to form 15 preliminary sub-themes. The detailed analysis yielded six main themes across varying contexts on self-management of diabetes and barriers to self-management. These six themes are presented in Figure 3 and discussed in Table 2 in detail providing the opinions of GP’s and nurses based on the various themes.

### 3.1. Low Self-Efficacy

The analysis revealed that some of the GP’s were personally affected by conflicts with their patients such as having feelings of frustration or blaming the patients for not listening to them. Nurses reflected the same feelings suggesting that lack of follow-up of GP’s advice have frustrated them. In addition, the GP’s inability to change the approach of patients undermined their confidence as a doctor which may lead to them “giving up” hope in their efforts to help these patients.

“*I consider the non-compliance of patients to the advice given to them as a rude behaviour on their part”. “I came to this part of the country with great enthusiasm to help this under-served population and then feel frustrated when realize that I am talking to the walls and then the enthusiasm for putting the patient on the right track diminishes with the time.*”

(GP-1)

“*I always asked them to follow the GP’s advice but patients lack of follow-up of GP’s advice all the time frustrated me*”

(Nurse-2)

However, other GP’s expressed hope and determination to sort out the problems by involving patients more and making themselves more available to patients.

“*I have told patients, in case they have any problems in managing their diabetes or they face any problem or need any clarifications, just call me and I will help them out even after working hours.*”

(GP-5)

These GP’s claimed that by helping the patients that way, they have been able to develop some sort of strategies to take care of conflicts they have with poorly controlled diabetes patients. On the contrary, other GP’s perceived themselves as “victims of patients” who prevented them from exercising their medical expertise.

“*I have received a call from the patient that he has a problem controlling his sugar level in the morning (fasting glucose level > 140 mg/dl). I advised him to have light meals in the night, take prescribed medicines, walk for 30 minutes and have at least 3 hours gap between the food and sleep and that worked very well for the patient.*”

(GP-8)

“*After couple of days, the patient sent me a message on my mobile thanking me for the help and advice. I think that kind of communication may bridge the gap of misunderstanding between the patient and doctor.*”

(GP-8)

It seems that patients prefer to have that type of relationship, that is a patient-centred approach—they feel comfortable with that type of approach.

“*I always discussed a lot with the patients about their self-management activities and encouraged them to continue or make more efforts – that way patients are encouraged and felt that they are taken care of the clinical staff.*”

(Nurse-3)

It appears with these discussions that some GP’s have been able to develop strategies to overcome the conflicts they have with poorly controlled diabetes patients. While a doctor–patient relationship was realized in those cases, however, it was not possible to establish an effective doctor–patient relationship in some cases. A relationship of understanding between the GP’s and patients was established to some extent. However, there was still some resistance with regards to “shared decision making” and a lack of mutual understanding.

### 3.2. Influence on Diabetes Self-Management Activities

The GP’s and nurses spoke about the differences in the experiences of self-management of diabetes between men and women. Our findings showed that the structure of gender roles within the family often meant that in comparison to men, women’s effort to “self-manage” were less likely to be supported by children and male partners, who were often unwilling to adopt diabetic-friendly diets.

“*Women in this rural area of Pakistan had a difficult time in managing their diabetes as compared to men. In this society, women cook the food according to the choices of the family–women don’t have much to say on the choice of the food, so they have no idea how to manage their diabetes in the environment they live and in relation to the healthy food choices.*”

(GP-6)

One of the healthcare nurses described the pressures that occurred on special occasions:

“*In Pakistani culture, if unhealthy food is served in parties on a special occasion, it is considered rude not to eat that and bringing diabetes–appropriate food to such events would not be accepted.*”

(Nurse-4)

A lack of understanding of diabetes self-management existed in people of Pakistan which might have posed barriers to the activities related to self-management of diabetes. Participants also reported that there was a strong moral aspect to self-management with deteriorating health due to diabetes being linked to a failing self, in particular a failure of self-control.

The GPs described the behaviour of patients in relation to diabetes management, suggesting that some patients showed insufficient self-efficacy and instead relied heavily on God’s Will and the destiny. This showed that this population relied heavily on the religious belief to cure their diabetes.

“*Religious belief plays an important role in this population. Some patients considered that this disease came from “Allah” (God), so Allah will cure that as well so no need to make efforts on self-management activities.*”

(GP-10)

One of the nurses mentioned that in informal discussions with some patients, particularly the male patients, they exhibited less interest than others in following instructions on diet and physical activities, and had fatalistic attitudes.

“*One of the nurses mentioned that patients are reluctant to follow the strict diet and want to enjoy the food of their choices.*”

(Nurse-5)

On the question of improving the diabetes care of patients with diabetes, GPs and nurses were of the opinion that there was the need for education to the patients on self-management and self-monitoring behaviour.

“*There is a need to promote self-monitoring behaviour and health education for the patients as these patients do not understand the complications of the disease and how to care for themselves.*”

(GP-9)

“*I think that diabetes self-care education can play an important role for this population as there is a complete lack of knowledge about this disease among these patients.*”

(Nurse-6)

### 3.3. Patient-Doctor Relationship

The general practitioners expressed disappointment in relation to their patients’ adherence with treatment and their approach towards the self-management activities.

“*I have provided clear direction how to use the medicines and follow up the healthy diet and exercise to my patients but it was of no use as the patients did not follow my advice. In fact, patients complained that there was no control on blood sugar and indicated that their health further deteriorated.*”

(GP-10)

The GPs reported that some patients delegated full responsibility for control of their diabetes to the GP and accepted none themselves.

“*The patients do not follow my suggestions, and hence, they decided not to adhere to dietary and behavioural recommendations.*”

(GP-10)

The general practitioners discussed how some diabetes patients had wrong concepts of self-management, opting to use traditional medicines without informing their doctors.

“*…. Many patients in Pakistan are using traditional medicines and sometime their side effects make them more sick blaming the general practitioners for not looking after their health well.*”

(GP-7)

The GPs claimed that they do what is best for their patients, but have a difficult time understanding why their advice was not properly followed by the patients. The nurses were of the opinion that they expected that patients would follow-up doctor’s advice, but this never happened. Most of the GPs and nurses reported that very few of their patients were following their diabetes management plan as explained to them.

### 3.4. Non-Adherence to Diet and Exercise

All the participating GPs and nurses were of the opinion that self-management practices were almost absent from the middle-aged population of Pakistan living in rural areas. In particular, they reported that these patients lacked any concept that diet and exercise were an important part of their management. However, GPs mentioned that some of their patients spoke about the importance of physical activity and followed this. The lack of physical activity and poor eating habits were influenced by the culture, tradition and lifestyle behaviour of the people in the rural area of Pakistan.

“*There is no diet and exercise consideration—patients with diabetes eat whatever is cooked at home for the family. Physical activity is non-existent as they don’t have proper facilities in this area where they can safely do the physical activities.*”

(GP-1)

In Pakistani society, changes to diet and physical activity are constrained by the extended families living together (joint family living system) [39]. Adherence to self-management of diabetes is also affected by the resources available for the management [40]. For instance, healthy eating patterns and physical activity levels are not likely to occur or be maintained without convenient sources of healthy foods and attractive, safe settings. Thus, the social context is a significant factor in the access to key resources for self-management [40].

### 3.5. Conflicts with Patients

The general practitioners spoke about the on-going conflicts with the patients. They were of the opinion that some diabetes patients were difficult to convince of the objectives of diabetes treatment or the importance of self-management activities. Therefore, they do not agree with treatment plans resulting in poor management of diabetes.

“*In Pakistani society, food is considered a very important factor which unites people and keeps them together. Many patients with type 2 diabetes in Pakistan eat whatever is presented to them in parties, so the doctor’s advice is not followed.*”

(GP-2)

“*This type of behaviour always results in a conflict with the patients.*”

(GP-3)

The GPs expectations were that patients would change their behaviour. However, they knew that it was unlikely to happen as patient’s behaviour was often associated with the lack of understandings or mistrust. They mentioned that taking care of such patients was a futile exercise.

### 3.6. Lack of Support

It was revealed after discussing with the nurses that there was a lack of support from the healthcare providers, spouse, and family members for the patients of diabetes. Nurses mentioned that patients told them that they did not obtain any help from the family or healthcare providers in managing their diabetes.

“*Patients don’t get any support from the family members to manage their diabetes. It was hard for them to cook diabetes “health-food” which may be separate from the rest of the extended family members living together.*”

(Nurse-7)

During the interviews, the general practitioners and nurses showed their frustration with the overall behaviour of patients with diabetes and their approach towards diabetes self-management activities. The analysis revealed that GPs considered giving up the struggle with their patients and at some stage may have been willing to accept whatever their patients were doing to manage their diabetes.

“*I feel like giving up my efforts as there is no way I can convince these patients about the complications of diabetes and make them understand the benefits of self-management of diabetes.*”

(GP-2)

However, although they reported being resigned to failure, they still adhered to their normal diabetes plan of action with a hope that one day they might be able to influence these patients to comply with the diabetes self-management activities and properly follow the GP’s advice given to them.

## 4. Discussion

The qualitative design of this study was guided by a methodological framework approach and the results of the systematic review of self-management of type 2 diabetes [23,24]. This framework described the healthcare professionals’ perspectives of type 2 diabetes patients and identified six major themes. With a qualitative methodological framework approach (used during interviews of healthcare professionals), this study aimed to help understanding the healthcare professional’s perspectives of patients with type 2 diabetes, experiencing difficulties with self-management activities and the barriers and challenges diabetes self-management is inflicting on these patients. This in turn will help inform effects to achieve better overall management of type 2 diabetes and the health outcomes for patients in rural Pakistan. The results will be relevant for changing clinical practice, constructing educational interventions, and composing guidelines, as these will benefit from reflecting and considering GPs’ attitudes towards diabetes patients.

GPs and nurses expressed frustration with the attitudes of their patients and lack of adherence to care plans. They reported that some patients refused to accept responsibility for self-management in relation to diet. This brought the general practitioners into conflict with their patients with some GPs resigning themselves to poor control of the diabetes of their patients as a result. In a descriptive qualitative study in Belgium, Wens et al. [41] reported similar feelings of frustration by doctors because their patients for whom diabetes medication was prescribed were poor compliers with treatment, including both oral medication and insulin.

However, other GPs mentioned that they used a patient-centred approach to resolve problems by being more involved with their patients and allowing them to contact them even after working hours in case patients needed their support and advice. These GPs claimed that by helping the patients that way, they had been able to overcome the challenges of poorly controlled diabetes patients having difficulties in managing their diabetes.

This kind of patient-centred approach by general practitioners was also identified and successfully followed in a study by Heisler et al. [42]. This study was carried out in an academic medical centre in Michigan (USA). In this study, GPs reported that having discussed more content areas of diabetes self-management was associated with greater agreement by the patients on treatment strategies. In our analysis, it was also revealed and acknowledged by GP’s that patients with type 2 diabetes preferred a patient-centred approach. However, other qualitative researchers found that the patient-centred approach was not easy for general practitioners to follow [43,44].

The factors influencing diabetes self-management included the social and cultural barriers which may have had a direct implication for self-management of type 2 diabetes. GPs agreed that diabetes self-management had a gendered dimension and women found it difficult to manage their diabetes as they did not have much to say on the choices of the food they cooked at home as food choices were dictated by other family members [45].

Rehan and Naz [46] investigated gender differences in diabetes self-management of type 2 diabetes in the province of Punjab, Pakistan. They have found that in Pakistani culture, food played an important role in uniting families. However, women reported a high emotional burden and distress while managing their diabetes, as food preparation brought them into conflict with the choices of family members. The importance of the relative positions of men and women was also acknowledged by Whittemore et al. [47], suggesting that women had primary responsibility for preparing food served in the family.

The GPs reported that some patients of type 2 diabetes relied on “God’s Will” to cure their diabetes. This shows the important role of religion in this population and its impact on self-management of diabetes. A number of the Muslim Indian and Pakistani participants in a study conducted in Edinburgh (Scotland) by Lawton et al. [48] professed similar beliefs that “it is in Allah’s hands” to cure them. Broom and Whittaker [49] indicated that such beliefs undermine the efforts of health professionals in managing diabetes.

In discussing the “patient-doctor relationship”, most of the GPs were unhappy with the patients not following their recommendations. The GP’s main concern was that most of the patients regardless of the age or gender, did not practice physical activity. Victim blaming of patients by doctors has been previously described by Feudtner [50] who in response, suggested that an open discussion should emerge between the two parties.

Other studies carried out in Pakistan found that education and awareness were low among the patients with diabetes and the studies highlighted the different ways in which cultural understandings were drawn on by participants and how this affected their approach to diabetes self-management [18,19,20,21,22,23,24,25].

GPs and nurses reported that patients with diabetes lacked support from spouses and other family members. Nurses were of the opinion that lack of family support may be due to the extended families living together with their food choices and not supporting the diabetic “healthy food”. However, extended families living together may also be considered to be a barrier to dietary adherence and self-management of diabetes [51]. According to Brown and Hedges [52], there is evidence to show that a low level of efficacy, lack of social and community support may have direct and indirect effects on the diabetes self-management activities.

Study Limitations: This study was carried out with a small sample and with a selective sampling approach. As such although the study identified a range of views, but their frequency cannot be determined. In addition, we have found some issues during the translation of the interviews. For example, in answering the questions by GPs there was a clear sound of frustration. Probably that might be the reason that the translated quotes sound rather blaming the patients. It may be argued that GPs number of years in medical practice might have contributed to their frustration. The main objective of this study was not to draw a conclusion which could be generalized, but to explore the healthcare professionals’ perspectives of type 2 diabetes patient’s experiences of self-management of diabetes in the rural area of Pakistan. This limitation should also be considered while interpreting the results of the interviews and drawing the final conclusion.

## 5. Conclusions

The results of this qualitative analysis highlighted the frustration that the general practitioners and nurses feel in seeking to encourage their diabetes patients to follow their advice on diabetes self-management in the rural area of Pakistan. The findings of this study may help in developing training for primary care practitioners especially in the support they provide to the patients for their self-management activities.

The results of this qualitative research suggest a need to change professional behaviour and attitude to promote a patient-centred approach rather than one based on blame. It also emphasizes the importance of health professionals being aware of the cultural and religious influences and beliefs of their patients so that care can remain focused on addressing the needs of the patients and improving quality of care and health outcomes. That knowledge will also help health professionals to better understand the contextual determinants of behaviours for future development of culturally appropriate diabetes self-management education to influence the illness beliefs and support the self-management activities of patients with type 2 diabetes.

## 6. Practice Implication

- This study is the first of its kind to explore the healthcare professionals’ perspectives of type 2 diabetes mellitus patients in the middle-aged population of Pakistan.
- The article highlighted the ways type 2 diabetes is managed in Pakistan.
- This article will help to minimize the gap between patient–doctor relationships and to achieve optimal glycaemic control and medication adherence.
- Healthcare professionals to set achievable management and self-management goals.

## Figures and Tables

**Table 1:** The summary of participants’ demographics.

Demographic	GPs (*n* = 10)	Nurses (*n* = 10)	Total (*n* = 20)
**Age (average, in years)**	48	50	49
**Marital Status**			
Single/never married	0	1	1
Married	10	9	19
Separated/divorced	0	0	0
**Education**			
Completed college or university	10	10	20
Professional degree (MBBS/MD)	10	0	10
Specialization (MRCGP/MRCP)	4	0	4
**Employment**			
Full/part-time	10	10	20
Unemployed	0	0	0
**Years in Practice (mean)**	16	18	17
**Family Background**			
Languages spoken at home	Urdu/Punjabi Pushto	Urdu/Punjabi Pushto	-
Cultural Background	Mohajir/Punjabi and Pathan	Mohajir/Punjabi and Pathan	-

**Table 2:** The summary of six major themes from data analysis.

Themes	GP’s (Feelings)	Nurses (Feelings)
Low self-efficacy	Personally affected by conflicts with the patients	Lack of follow-up by patients frustrating
Influence on diabetes self-management activities	Social and cultural barriers	Barriers from: joint family, religious
Patient–doctor relationship	Conflict with the patients	More expectations from patients
Non-adherence to diet and exercise	Patients do not follow instructions	Difficulties to follow-up instructions
Conflicts with patients	Patients do not listen to advice	Patients poor attitude
Lack of support	No support from the Healthcare providers No Social support	Less support from family

## Data Availability

The data presented in this study are available on request from the corresponding author. The data are not publicly available due to restriction on data disclosure.

## Abbreviations

IDF: International Diabetes Federation; HbA1c: Glycosylated hemoglobin

## Appendix A

Qualitative interview guide for Healthcare Professionals

1. Please tell me about the barriers to self-management in patients? Are there any issues related to?
  - Human resources and the availability of trained staff
  - Funding for clinical consultations, medicines and other public health services
  - Cultural/religious barriers which might prevent people from seeking care
2. To what extent do diabetic patients use existing services?
  - What is your experience of under-usage?
  - What is your experience of over-usage?
  - What is the effect of the service usage patterns you have mentioned?
    *[Prompt: relevant to self-management]*
3. To what extent does culture influence patients’ access to health care?
  - What in particular influences the Pakistani communities?
  - Can you give me an example specific to the Pakistani communities?
4. Many people in Pakistan with chronic illness like diabetes often report seeking traditional medicines. What do you think about that? Are there any issues related to?
  - Reported effect of these treatments
  - Self-medication
  - Side-effects reported from the treatment
5. How do patients respond to the treatments you might prescribe? Are there any issues related to?
  - Reported effect of the treatment
  - Self-medication
  - Non-compliance
  - Side-effects reported from the treatment
6. Thinking about the issues raised in the earlier discussions, what could be done to address or overcome these issues?
7. What do you think would improve diabetes care of patients with type 2 diabetes?
  *[Prompt: what impacts would more self-management have?]*
8. What could be done in your profession to improve things?
9. What sort of things need to be done more broadly to improve service for people with diabetes?
10. Are there any other issues that you want to raise that we have not discussed about management of diabetes?

Thank you for your time and cooperation for this interview.
